# Supplementary material for: A Mediation Analysis to Identify Links between Gut Bacteria and Memory in Context of Human Milk Oligosaccharides
Source: Microorganisms. 2021 Apr 15;9(4):846. doi: 10.3390/microorganisms9040846 (PMC8071191; doi:10.3390/microorganisms9040846)
Supplement: Supplementary file 1 [file microorganisms-09-00846-s001.zip › microorganisms-1169300-supplementary.pdf]

Table S1. Variables included in the mediation analysis.

No	Variable	Category	Explanation
1	Acidaminococcus (AC)	16S ribosomal sequencing	Relative abundance of Acidaminococcus, % in the ascending colon
2	Akkermansia (AC)	16S ribosomal sequencing	Relative abundance of Akkermansia, % in the ascending colon
3	Alistipes (AC)	16S ribosomal sequencing	Relative abundance of Alistipes, % in the ascending colon
4	Anaerotruncus (AC)	16S ribosomal sequencing	Relative abundance of Anaerotruncus, % in the ascending colon
5	Anaerovibrio (AC)	16S ribosomal sequencing	Relative abundance of Anaerovibrio, % in the ascending colon
6	Bifidobacterium (AC)	16S ribosomal sequencing	Relative abundance of Bifidobacterium, % in the ascending colon
7	Bilophila (AC)	16S ribosomal sequencing	Relative abundance of Bilophila, % in the ascending colon
8	Blautia (AC)	16S ribosomal sequencing	Relative abundance of Blautia, % in the ascending colon
9	Butyricicoccus (AC)	16S ribosomal sequencing	Relative abundance of Butyricicoccus, % in the ascending colon
10	Butyricimonas (AC)	16S ribosomal sequencing	Relative abundance of Butyricimonas, % in the ascending colon
11	Campylobacter (AC)	16S ribosomal sequencing	Relative abundance of Campylobacter, % in the ascending colon
12	Catenibacterium (AC)	16S ribosomal sequencing	Relative abundance of Catenibacterium, % in the ascending colon
13	Clostridium (AC)	16S ribosomal sequencing	Relative abundance of Clostridium, % in the ascending colon
14	Collinsella (AC)	16S ribosomal sequencing	Relative abundance of Collinsella, % in the ascending colon
15	Coprococcus (AC)	16S ribosomal sequencing	Relative abundance of Coprococcus, % in the ascending colon
16	Desulfovibrio (AC)	16S ribosomal sequencing	Relative abundance of Desulfovibrio, % in the ascending colon
17	Dialister (AC)	16S ribosomal sequencing	Relative abundance of Dialister, % in the ascending colon
18	Dorea (AC)	16S ribosomal sequencing	Relative abundance of Dorea, % in the ascending colon
19	Escherichia (AC)	16S ribosomal sequencing	Relative abundance of Escherichia, % in the ascending colon
20	Eubacterium (AC)	16S ribosomal sequencing	Relative abundance of Eubacterium, % in the ascending colon
21	Faecalibacterium (AC)	16S ribosomal sequencing	Relative abundance of Faecalibacterium, % in the ascending colon
22	Flexispira (AC)	16S ribosomal sequencing	Relative abundance of Flexispira, % in the ascending colon
23	Lactobacillus (AC)	16S ribosomal sequencing	Relative abundance of Lactobacillus, % in the ascending colon
24	Lactococcus (AC)	16S ribosomal sequencing	Relative abundance of Lactococcus, % in the ascending colon
25	Leuconostoc (AC)	16S ribosomal sequencing	Relative abundance of Leuconostoc, % in the ascending colon
26	Megasphaera (AC)	16S ribosomal sequencing	Relative abundance of Megasphaera, % in the ascending colon
27	Mitsuokella (AC)	16S ribosomal sequencing	Relative abundance of Mitsuokella, % in the ascending colon
28	Mogibacterium (AC)	16S ribosomal sequencing	Relative abundance of Mogibacterium, % in the ascending colon
29	Mucispirillum (AC)	16S ribosomal sequencing	Relative abundance of Mucispirillum, % in the ascending colon
30	Oscillospira (AC)	16S ribosomal sequencing	Relative abundance of Oscillospira, % in the ascending colon
31	Parabacteroides (AC)	16S ribosomal sequencing	Relative abundance of Parabacteroides, % in the ascending colon
32	Pasteurella (AC)	16S ribosomal sequencing	Relative abundance of Pasteurella, % in the ascending colon
33	Peptococcus (AC)	16S ribosomal sequencing	Relative abundance of Peptococcus, % in the ascending colon
34	Phascolarctobacterium (AC)	16S ribosomal sequencing	Relative abundance of Phascolarctobacterium, % in the ascending colon
35	Prevotella (AC)	16S ribosomal sequencing	Relative abundance of Prevotella, % in the ascending colon
36	Prevotella (AC)	16S ribosomal sequencing	Relative abundance of Prevotella, % in the ascending colon
37	Pyramidobacter (AC)	16S ribosomal sequencing	Relative abundance of Pyramidobacter, % in the ascending colon
38	RFN20 (AC)	16S ribosomal sequencing	Relative abundance of RFN20, % in the ascending colon
39	Ruminococcus (AC)	16S ribosomal sequencing	Relative abundance of Ruminococcus, % in the ascending colon
40	Ruminococcus (AC)	16S ribosomal sequencing	Relative abundance of Ruminococcus, % in the ascending colon
41	Sharpea (AC)	16S ribosomal sequencing	Relative abundance of Sharpea, % in the ascending colon

Table S1. Variables included in the mediation analysis.

No	Variable	Category	Explanation
42	Streptococcus (AC)	16S ribosomal sequencing	Relative abundance of Streptococcus, % in the ascending colon
43	Sutterella (AC)	16S ribosomal sequencing	Relative abundance of Sutterella, % in the ascending colon
44	Synergistes (AC)	16S ribosomal sequencing	Relative abundance of Synergistes, % in the ascending colon
45	Unclassified (AC)	16S ribosomal sequencing	Relative abundance of , % in the ascending colon
46	Turicibacter (AC)	16S ribosomal sequencing	Relative abundance of Turicibacter, % in the ascending colon
47	Uncl. Coriobacteriaceae (AC)	16S ribosomal sequencing	Relative abundance of Uncl. Coriobacteriaceae, % in the ascending colon
48	Uncl. Desulfovibrionaceae (AC)	16S ribosomal sequencing	Relative abundance of Uncl. Desulfovibrionaceae, % in the ascending colon
49	Uncl. Elusimicrobiaceae (AC)	16S ribosomal sequencing	Relative abundance of Uncl. Elusimicrobiaceae, % in the ascending colon
50	Uncl. Enterobacteriaceae (AC)	16S ribosomal sequencing	Relative abundance of Uncl. Enterobacteriaceae, % in the ascending colon
51	Uncl. Enterococcaceae (AC)	16S ribosomal sequencing	Relative abundance of Uncl. Enterococcaceae, % in the ascending colon
52	Uncl. Erysipelotrichaceae (AC)	16S ribosomal sequencing	Relative abundance of Uncl. Erysipelotrichaceae, % in the ascending colon
53	Uncl. Fusobacteriaceae (AC)	16S ribosomal sequencing	Relative abundance of Uncl. Fusobacteriaceae, % in the ascending colon
54	Uncl. Lachnospiraceae (AC)	16S ribosomal sequencing	Relative abundance of Uncl. Lachnospiraceae, % in the ascending colon
55	Uncl. Lactobacillaceae (AC)	16S ribosomal sequencing	Relative abundance of Uncl. Lactobacillaceae, % in the ascending colon
56	Uncl. Leuconostocaceae (AC)	16S ribosomal sequencing	Relative abundance of Uncl. Leuconostocaceae, % in the ascending colon
57	Uncl. Mogibacteriaceae (AC)	16S ribosomal sequencing	Relative abundance of Uncl. Mogibacteriaceae, % in the ascending colon
58	Uncl. Paraprevotellaceae (AC)	16S ribosomal sequencing	Relative abundance of Uncl. Paraprevotellaceae, % in the ascending colon
59	Uncl. Prevotellaceae (AC)	16S ribosomal sequencing	Relative abundance of Uncl. Prevotellaceae, % in the ascending colon
60	Uncl. Rikenellaceae (AC)	16S ribosomal sequencing	Relative abundance of Uncl. Rikenellaceae, % in the ascending colon
61	Uncl. Ruminococcaceae (AC)	16S ribosomal sequencing	Relative abundance of Uncl. Ruminococcaceae, % in the ascending colon
62	Uncl. S24-7 (AC)	16S ribosomal sequencing	Relative abundance of Uncl. S24-7, % in the ascending colon
63	Uncl. Veillonellaceae (AC)	16S ribosomal sequencing	Relative abundance of Uncl. Veillonellaceae, % in the ascending colon
64	Acidaminococcus (F)	16S ribosomal sequencing	Relative abundance of Acidaminococcus, % in feces
65	Akkermansia (F)	16S ribosomal sequencing	Relative abundance of Akkermansia, % in feces
66	Alistipes (F)	16S ribosomal sequencing	Relative abundance of Alistipes, % in feces
67	Anaerotruncus (F)	16S ribosomal sequencing	Relative abundance of Anaerotruncus, % in feces
68	Bacteroides (F)	16S ribosomal sequencing	Relative abundance of Bacteroides, % in feces
69	Bifidobacterium (F)	16S ribosomal sequencing	Relative abundance of Bifidobacterium, % in feces
70	Bilophila (F)	16S ribosomal sequencing	Relative abundance of Bilophila, % in feces
71	Blautia (F)	16S ribosomal sequencing	Relative abundance of Blautia, % in feces
72	Butyricicoccus (F)	16S ribosomal sequencing	Relative abundance of Butyricicoccus, % in feces
73	Butyricimonas (F)	16S ribosomal sequencing	Relative abundance of Butyricimonas, % in feces
74	Campylobacter (F)	16S ribosomal sequencing	Relative abundance of Campylobacter, % in feces
75	Catenibacterium (F)	16S ribosomal sequencing	Relative abundance of Catenibacterium, % in feces
76	Christensenella (F)	16S ribosomal sequencing	Relative abundance of Christensenella, % in feces
77	Clostridium (F)	16S ribosomal sequencing	Relative abundance of Clostridium, % in feces
78	Clostridium (F)	16S ribosomal sequencing	Relative abundance of Clostridium, % in feces
79	Collinsella (F)	16S ribosomal sequencing	Relative abundance of Collinsella, % in feces
80	Coprococcus (F)	16S ribosomal sequencing	Relative abundance of Coprococcus, % in feces
81	Desulfovibrio (F)	16S ribosomal sequencing	Relative abundance of Desulfovibrio, % in feces
82	Dialister (F)	16S ribosomal sequencing	Relative abundance of Dialister, % in feces

Table S1. Variables included in the mediation analysis.

No	Variable	Category	Explanation
83	Dorea (F)	16S ribosomal sequencing	Relative abundance of Dorea, % in feces
84	Escherichia (F)	16S ribosomal sequencing	Relative abundance of Escherichia, % in feces
85	Eubacterium (F)	16S ribosomal sequencing	Relative abundance of Eubacterium, % in feces
86	Faecalibacterium (F)	16S ribosomal sequencing	Relative abundance of Faecalibacterium, % in feces
87	Flexispira (F)	16S ribosomal sequencing	Relative abundance of Flexispira, % in feces
88	Lactobacillus (F)	16S ribosomal sequencing	Relative abundance of Lactobacillus, % in feces
89	Lactococcus (F)	16S ribosomal sequencing	Relative abundance of Lactococcus, % in feces
90	Leuconostoc (F)	16S ribosomal sequencing	Relative abundance of Leuconostoc, % in feces
91	Megasphaera (F)	16S ribosomal sequencing	Relative abundance of Megasphaera, % in feces
92	Mitsuokella (F)	16S ribosomal sequencing	Relative abundance of Mitsuokella, % in feces
93	Oscillospira (F)	16S ribosomal sequencing	Relative abundance of Oscillospira, % in feces
94	p-75-a5 (F)	16S ribosomal sequencing	Relative abundance of p-75-a5, % in feces
95	Parabacteroides (F)	16S ribosomal sequencing	Relative abundance of Parabacteroides, % in feces
96	Phascolarctobacterium (F)	16S ribosomal sequencing	Relative abundance of Phascolarctobacterium, % in feces
97	Prevotella (F)	16S ribosomal sequencing	Relative abundance of Prevotella, % in feces
98	Prevotella (F)	16S ribosomal sequencing	Relative abundance of Prevotella, % in feces
99	RFN20 (F)	16S ribosomal sequencing	Relative abundance of RFN20, % in feces
100	Ruminococcus (F)	16S ribosomal sequencing	Relative abundance of Ruminococcus, % in feces
101	Ruminococcus (F)	16S ribosomal sequencing	Relative abundance of Ruminococcus, % in feces
102	Sharpea (F)	16S ribosomal sequencing	Relative abundance of Sharpea, % in feces
103	Succiniclasticum (F)	16S ribosomal sequencing	Relative abundance of Succiniclasticum, % in feces
104	Sutterella (F)	16S ribosomal sequencing	Relative abundance of Sutterella, % in feces
105	Synergistes (F)	16S ribosomal sequencing	Relative abundance of Synergistes, % in feces
106	Turicibacter (F)	16S ribosomal sequencing	Relative abundance of Turicibacter, % in feces
107	Uncl. Barnesiellaceae (F)	16S ribosomal sequencing	Relative abundance of Uncl. Barnesiellaceae, % in feces
108	Uncl. Christensenellaceae (F)	16S ribosomal sequencing	Relative abundance of Uncl. Christensenellaceae, % in feces
109	Uncl. Coriobacteriaceae (F)	16S ribosomal sequencing	Relative abundance of Uncl. Coriobacteriaceae, % in feces
110	Uncl. Desulfovibrionaceae (F)	16S ribosomal sequencing	Relative abundance of Uncl. Desulfovibrionaceae, % in feces
111	Uncl. Elusimicrobiaceae (F)	16S ribosomal sequencing	Relative abundance of Uncl. Elusimicrobiaceae, % in feces
112	Uncl. Enterobacteriaceae (F)	16S ribosomal sequencing	Relative abundance of Uncl. Enterobacteriaceae, % in feces
113	Uncl. Erysipelotrichaceae (F)	16S ribosomal sequencing	Relative abundance of Uncl. Erysipelotrichaceae, % in feces
114	Uncl. Fusobacteriaceae (F)	16S ribosomal sequencing	Relative abundance of Uncl. Fusobacteriaceae, % in feces
115	Uncl. Lachnospiraceae (F)	16S ribosomal sequencing	Relative abundance of Uncl. Lachnospiraceae, % in feces
116	Uncl. Lactobacillaceae (F)	16S ribosomal sequencing	Relative abundance of Uncl. Lactobacillaceae, % in feces
117	Uncl. Mogibacteriaceae (F)	16S ribosomal sequencing	Relative abundance of Uncl. Mogibacteriaceae, % in feces
118	Uncl. Paraprevotellaceae (F)	16S ribosomal sequencing	Relative abundance of Uncl. Paraprevotellaceae, % in feces
119	Uncl. Peptostreptococcaceae (F)	16S ribosomal sequencing	Relative abundance of Uncl. Peptostreptococcaceae, % in feces
120	Uncl. Prevotellaceae (F)	16S ribosomal sequencing	Relative abundance of Uncl. Prevotellaceae, % in feces
121	Uncl. Rikenellaceae (F)	16S ribosomal sequencing	Relative abundance of Uncl. Rikenellaceae, % in feces
122	Uncl. Ruminococcaceae (F)	16S ribosomal sequencing	Relative abundance of Uncl. Ruminococcaceae, % in feces
123	Uncl. S24-7 (F)	16S ribosomal sequencing	Relative abundance of Uncl. S24-7, % in feces

Table S1. Variables included in the mediation analysis.

No	Variable	Category	Explanation
124	Uncl. Synergistaceae (F)	16S ribosomal sequencing	Relative abundance of Uncl. Synergistaceae, % in feces
125	Uncl. Veillonellaceae (F)	16S ribosomal sequencing	Relative abundance of Uncl. Veillonellaceae, % in feces
126	Uncl. Victivallaceae (F)	16S ribosomal sequencing	Relative abundance of Uncl. Victivallaceae, % in feces
127	Latency to first visit (1h)	Behavior	Latency to first visit to either object during the NOR test trial with a 1-hour delay, s
128	Latency to first visit (48h)	Behavior	Latency to first visit to either object during the NOR test trial with a 48-hour delay, s
129	Mean visit time (1h)	Behavior	Mean visit time to both objects during the NOR test trial with a 1-hour delay, s/visit
130	Mean visit time (48h)	Behavior	Mean visit time to both objects during the NOR test trial with a 48-hour delay, s/visit
131	Nov ave visit time (1h)	Behavior	Mean visit time to the novel object during the NOR test trial with a 1-hour delay, s/visit
132	Nov ave visit time (48h)	Behavior	Mean visit time to the novel object during the NOR test trial with a 48-hour delay, s/visit
133	Nov latency to first visit (1h)	Behavior	Latency to the first visit to the novel object during the NOR test trial with a 1-hour delay, s
134	Nov latency to first visit (48h)	Behavior	Latency to the first visit to the novel object during the NOR test trial with a 48-hour delay, s
135	Nov no. of visits (1h)	Behavior	Total number of visits to the novel object during the NOR test trial with a 1-hour delay
136	Nov no. of visits (48h)	Behavior	Total number of visits to the novel object during the NOR test trial with a 48-hour delay
137	Nov visit time (1h)	Behavior	Total time visiting the novel object during the NOR test trial with a 1-hour delay, s
138	Nov visit time (48h)	Behavior	Total time visiting the novel object during the NOR test trial with a 48-hour delay, s
139	Perimeter.p (1h)	Behavior	% time spent in the perimeter of the arena during the NOR test trial with a 1-hour delay
140	Perimeter.p (48h)	Behavior	% time spent in the perimeter of the arena during the NOR test trial with a 48-hour delay
141	RI (1h)	Behavior	Recognition Index, the time spent visiting the novel object divided by total time spent visiting both objects.
142	RI (48h)	Behavior	Recognition Index, the time spent visiting the novel object divided by total time spent visiting both objects.
143	Sam ave visit time (1h)	Behavior	Mean visit time to the sample object during the NOR test trial with a 1-hour delay, s/visit
144	Sam ave visit time (48h)	Behavior	Mean visit time to the sample object during the NOR test trial with a 48-hour delay, s/visit
145	Sam latency to first visit (1h)	Behavior	Latency to the first visit to the sample object during the NOR test trial with a 1-hour delay, s
146	Sam latency to first visit (48h)	Behavior	Latency to the first visit to the sample object during the NOR test trial with a 48-hour delay, s
147	Sam no. of visits (1h)	Behavior	Total number of visits to the sample object during the NOR test trial with a 1-hour delay
148	Sam no. of visits (48h)	Behavior	Total number of visits to the sample object during the NOR test trial with a 48-hour delay
149	Sam visit time (1h)	Behavior	Total time visiting the sample object during the NOR test trial with a 1-hour delay, s
150	Sam visit time (48h)	Behavior	Total time visiting the sample object during the NOR test trial with a 48-hour delay, s
151	Total dis. moved (1h)	Behavior	Total distance moved during the NOR test trial with a 1-hour delay, cm
152	Total dis. moved (48h)	Behavior	Total distance moved during the NOR test trial with a 48-hour delay, cm
153	Total no. of visits (1h)	Behavior	Total number of visits to both objects during the NOR test trial with a 1-hour delay
154	Total no. of visits (48h)	Behavior	Total number of visits to both objects during the NOR test trial with a 48-hour delay
155	Total visit time (1h)	Behavior	Total time visiting both objects during the NOR test trial with a 1-hour delay, s
156	Total visit time (48h)	Behavior	Total time visiting both objects during the NOR test trial with a 48-hour delay, s
157	*BDNF*	Gene Expression	Brain Derived Neurotrophic Factor
158	*C-FOS*	Gene Expression	Proto-Oncogene C-Fos, an immediate early gene
159	*CHRM1*	Gene Expression	Cholinergic Receptor Muscarinic 1
160	*CHRM2*	Gene Expression	Cholinergic Receptor Muscarinic 2
161	*CHRM3*	Gene Expression	Cholinergic Receptor Muscarinic 3
162	*CHRM5*	Gene Expression	Cholinergic Receptor Muscarinic 5
163	*CHRNA2*	Gene Expression	Cholinergic Receptor Nicotinic Alpha 2
164	*CHRNA7*	Gene Expression	Cholinergic Receptor Nicotinic Alpha 7

Table S1. Variables included in the mediation analysis.

No	Variable	Category	Explanation
165	*CHRNB4*	Gene Expression	Cholinergic Receptor Nicotinic Beta 4
166	*CREB*	Gene Expression	cyclic AMP Response Element Binding Protein
167	*CREBBP*	Gene Expression	CREB Binding Protein
168	*DLG4*	Gene Expression	Discs Large MAGUK Scaffold Protein, aka postsynaptic density protein 95
169	*EGR1*	Gene Expression	Early Growth Response 1
170	*GABBR1*	Gene Expression	GABA B Receptor Subunit 1
171	*GABRA1*	Gene Expression	GABA Receptor Type A Alpha 1
172	*GABRA2*	Gene Expression	GABA Receptor Type A Alpha 2
173	*GABRA5*	Gene Expression	GABA Receptor Type A Alpha 5
174	*GABRB2*	Gene Expression	GABA Type A Receptor Beta 2
175	*GABRD*	Gene Expression	GABA Type A Receptor Delta Subunit
176	*GABRG2*	Gene Expression	GABA Type A Receptor Gamma 2 Subunit
177	*GABRR1*	Gene Expression	GABA Type A Receptor Rho 1 Subunit
178	*GAD*	Gene Expression	Glutamate Decarboxylase
179	*GLRA1*	Gene Expression	Glycine Receptor Alpha 1
180	*GLRA2*	Gene Expression	Glycine Receptor Alpha 2
181	*GLRA3*	Gene Expression	Glycine Receptor Alpha 3
182	*GRIA1*	Gene Expression	Glutamate Ionotropic Receptor AMPA Type Subunit 1
183	*GRIA2*	Gene Expression	Glutamate Ionotropic Receptor AMPA Type Subunit 2
184	*GRIA3*	Gene Expression	Glutamate Ionotropic Receptor AMPA Type Subunit 3
185	*GRIA4*	Gene Expression	Glutamate Ionotropic Receptor AMPA Type Subunit 4
186	*GRIN1*	Gene Expression	Glutamate Ionotropic Receptor NMDA Type Subunit 1
187	*GRIN2A*	Gene Expression	Glutamate Ionotropic Receptor NMDA Type Subunit 2A
188	*GRIN2B*	Gene Expression	Glutamate Ionotropic Receptor NMDA Type Subunit 2B
189	*GRIN2D*	Gene Expression	Glutamate Ionotropic Receptor NMDA Type Subunit 2D
190	*HDAC1*	Gene Expression	Histone Deacetylase 1
191	*HDAC2*	Gene Expression	Histone Deacetylase 2
192	*HDAC3*	Gene Expression	Histone Deacetylase 3
193	*HDAC4*	Gene Expression	Histone Deacetylase 4
194	*HDAC5*	Gene Expression	Histone Deacetylase 5
195	*HDAC7*	Gene Expression	Histone Deacetylase 7
196	*HDAC8*	Gene Expression	Histone Deacetylase 8
197	*HDAC9*	Gene Expression	Histone Deacetylase 9
198	*HOMER1*	Gene Expression	Homer Scaffold Protein 1
199	*IGF1*	Gene Expression	Insulin Like Growth Factor 1
200	*IGF2*	Gene Expression	Insulin Like Growth Factor 2
201	*MAG*	Gene Expression	Myelin Associated Glycoprotein
202	*MBP*	Gene Expression	Myelin Basic Protein
203	*NCAM1*	Gene Expression	Neural Cell Adhesion Molecule 1
204	*NPY*	Gene Expression	Neuropeptide Y
205	*NR3C1*	Gene Expression	Glucocorticoid Nuclear Receptor Variant 1

Table S1. Variables included in the mediation analysis.

No	Variable	Category	Explanation
206	*NR3C2*	Gene Expression	Mineralocorticoid Receptor
207	*NR4A1*	Gene Expression	Nerve growth factor IB Nuclear Receptor Variant 1
208	*NR4A2*	Gene Expression	Orphan Nuclear Receptor NURR1
209	*PLP*	Gene Expression	Proteolipid Protein 1
210	*PP1AC*	Gene Expression	Protein Phosphatase 1
211	*SIRT1*	Gene Expression	Sirtuin 1
212	*SLC17A6*	Gene Expression	Vesicular Glutamate Transporter 2
213	*SLC17A7*	Gene Expression	Vesicular Glutamate Transporter 1
214	*SLC17A8*	Gene Expression	Vesicular Glutamate Transporter 3
215	*SLC1A1*	Gene Expression	Excitatory Amino-Acid Transporter 3
216	*SLC1A2*	Gene Expression	Excitatory Amino-Acid Transporter 2
217	*SLC1A3*	Gene Expression	Excitatory Amino-Acid Transporter 1
218	*SLC1A6*	Gene Expression	Excitatory Amino-Acid Transporter 4
219	*SLC32A1*	Gene Expression	Vesicular GABA Transporter
220	*SLC6A1*	Gene Expression	Sodium- and Chloride-Dependent GABA Transporter 1
221	*SLC6A11*	Gene Expression	Sodium- and Chloride-Dependent GABA Transporter 3
222	*SLC6A13*	Gene Expression	Sodium- and Chloride-Dependent GABA Transporter 2
223	*SNAP25*	Gene Expression	Synaptosome Associated Protein 25
224	*SYP*	Gene Expression	Synaptophysin
225	*UBE3A*	Gene Expression	Ubiquitin Protein Ligase E3A
226	*5-HTR1*	Gene Expression	5-Hydroxytryptamine Receptor 1
227	*5-HTR2*	Gene Expression	5-Hydroxytryptamine Receptor 2
228	*5-HTR4*	Gene Expression	5-Hydroxytryptamine Receptor 4
229	*5-HTR7*	Gene Expression	5-Hydroxytryptamine Receptor 7
230	GABA	Magnetic resonance imaging	Single-Voxel Spectroscopy analysis of GABA, ppm
231	Glutathione	Magnetic resonance imaging	Single-Voxel Spectroscopy analysis of Glutathione, ppm
232	Inositol	Magnetic resonance imaging	Single-Voxel Spectroscopy analysis of Inositol, ppm
233	N-acetylaspartate	Magnetic resonance imaging	Single-Voxel Spectroscopy analysis of N-acetylaspartate, ppm
234	faCaudate	Magnetic resonance imaging	Fractional Anisotropy of the Caudate
235	faCorpus C.	Magnetic resonance imaging	Fractional Anisotropy of the Corpus Callosum
236	faCerebellum	Magnetic resonance imaging	Fractional Anisotropy of the Cerebellum
237	faInternal C.	Magnetic resonance imaging	Fractional Anisotropy of the Internal Capsule
238	faLeft Crtx	Magnetic resonance imaging	Fractional Anisotropy of the Left Cortex
239	faLeft Hip.	Magnetic resonance imaging	Fractional Anisotropy of the Left Hippocampus
240	faRight Crtx	Magnetic resonance imaging	Fractional Anisotropy of the Right Cortex
241	faRight Hip.	Magnetic resonance imaging	Fractional Anisotropy of the Right Hippocampus
242	faThalamus	Magnetic resonance imaging	Fractional Anisotropy of the Thalamus
243	faWhole B.	Magnetic resonance imaging	Fractional Anisotropy of the Whole Brain
244	faWhite	Magnetic resonance imaging	Fractional Anisotropy of the White Matter
245	Cerebral Aqueduct	Magnetic resonance imaging	Absolute Volume of the Cerebral Aqueduct, mm3
246	Caudate	Magnetic resonance imaging	Absolute Volume of the Caudate, mm3

Table S1. Variables included in the mediation analysis.

No	Variable	Category	Explanation
247	Corpus C.	Magnetic resonance imaging	Absolute Volume of the Corpus Callosum, mm3
248	Cerebellum	Magnetic resonance imaging	Absolute Volume of the Cerebellum, mm3
249	CSF	Magnetic resonance imaging	Absolute Volume of Cerebrospinal Fluid, mm3
250	Fourth Vent.	Magnetic resonance imaging	Absolute Volume of the Fourth Ventricle, mm3
251	Grey M.	Magnetic resonance imaging	Absolute Volume of Grey Matter, mm3
252	Hypothalamus	Magnetic resonance imaging	Absolute Volume of the Hypothalamus, mm3
253	Internal C.	Magnetic resonance imaging	Absolute Volume of the Internal Capsule, mm3
254	Left Crtx	Magnetic resonance imaging	Absolute Volume of the Left Cortex, mm3
255	Left Hip.	Magnetic resonance imaging	Absolute Volume of the Left Hippocampus, mm3
256	Lateral Vent.	Magnetic resonance imaging	Absolute Volume of the Lateral Ventricle, mm3
257	Medul	Magnetic resonance imaging	Absolute Volume of the Medulla, mm3
258	Midbr	Magnetic resonance imaging	Absolute Volume of the Midbrain, mm3
259	OlfBul	Magnetic resonance imaging	Absolute Volume of the Olfactory Bulb, mm3
260	Pons	Magnetic resonance imaging	Absolute Volume of the Pons, mm3
261	PutGP	Magnetic resonance imaging	Absolute Volume of the Putamen Globus Pallidus, mm3
262	Right Crtx	Magnetic resonance imaging	Absolute Volume of the Right Cortex, mm3
263	Right Hip.	Magnetic resonance imaging	Absolute Volume of the Right Hippocampus, mm3
264	Thalamus	Magnetic resonance imaging	Absolute Volume of the Thalamus, mm3
265	Third Vent.	Magnetic resonance imaging	Absolute Volume of the Third Ventricle, mm3
266	Whole B.	Magnetic resonance imaging	Absolute Volume of the Whole Brain, mm3
267	White M.	Magnetic resonance imaging	Absolute Volume of White Matter, mm3
268	Rel. Cer. Aq.	Magnetic resonance imaging	Relative Volume of the Cerebral Aqueduct, %TBV
269	Rel. Caudate	Magnetic resonance imaging	Relative Volume of the Caudate, %TBV
270	Rel. Corpus C.	Magnetic resonance imaging	Relative Volume of the Corpus Callosum, %TBV
271	Rel. Cerebellum	Magnetic resonance imaging	Relative Volume of the Cerebellum, %TBV
272	Rel. CSF	Magnetic resonance imaging	Relative Volume of Cerebrospinal Fluid, %TBV
273	Rel. FV	Magnetic resonance imaging	Relative Volume of the Fourth Ventricle, %TBV
274	Rel. Grey M.	Magnetic resonance imaging	Relative Volume of Grey Matter, %TBV
275	Rel. Hypothalamus	Magnetic resonance imaging	Relative Volume of the Hypothalamus, %TBV
276	Rel. Internal C.	Magnetic resonance imaging	Relative Volume of the Internal Capsule, %TBV
277	Rel. Left Crtx	Magnetic resonance imaging	Relative Volume of the Left Cortex, %TBV
278	Rel. Left Hip.	Magnetic resonance imaging	Relative Volume of the Left Hippocampus, %TBV
279	Rel. Lateral Vent.	Magnetic resonance imaging	Relative Volume of the Lateral Ventricle, %TBV
280	Rel. Medulla	Magnetic resonance imaging	Relative Volume of the Medulla, %TBV
281	Rel. Midbrain	Magnetic resonance imaging	Relative Volume of the Midbrain, %TBV
282	Rel. OlfBul	Magnetic resonance imaging	Relative Volume of the Olfactory Bulb, %TBV
283	Rel. Pons	Magnetic resonance imaging	Relative Volume of the Pons, %TBV
284	Rel. PutGP	Magnetic resonance imaging	Relative Volume of the Putamen Globus Pallidus, %TBV
285	Rel. Right Crtx	Magnetic resonance imaging	Relative Volume of the Right Cortex, %TBV
286	Rel. Right Hip.	Magnetic resonance imaging	Relative Volume of the Right Hippocampus, %TBV
287	Rel. Thalamus	Magnetic resonance imaging	Relative Volume of the Thalamus, %TBV

Table S1. Variables included in the mediation analysis.

No	Variable	Category	Explanation
288	Rel. Third Vent.	Magnetic resonance imaging	Relative Volume of the Third Ventricle, %TBV
289	Rel. White M.	Magnetic resonance imaging	Relative Volume of White Matter, %TBV

Table S2. Sample size and missingness.

No	Variable	Sample Size[1]						Not-quantified observations (NQ)						Missing Observations[2]					
		CON	BMOS	BMOS + HMO	HMO	OF	OF + 2'FL	CON	BMOS	BMOS + HMO	HMO	OF	OF + 2'FL	CON	BMOS	BMOS + HMO	HMO	OF	OF + 2'FL
1	Acidaminococcus (AC)	10	12	10	12	12	11	0	0	0	0	0	0	2	0	0	0	0	1
2	Akkermansia (AC)	10	12	10	12	12	11	0	0	0	0	0	0	2	0	0	0	0	1
3	Alistipes (AC)	10	12	10	12	12	11	0	0	0	0	0	0	2	0	0	0	0	1
4	Anaerotruncus (AC)	10	12	10	12	12	11	0	0	0	0	0	0	2	0	0	0	0	1
5	Anaerovibrio (AC)	10	12	10	12	12	11	0	0	0	0	0	0	2	0	0	0	0	1
6	Bifidobacterium (AC)	10	12	10	12	12	11	0	0	0	0	0	0	2	0	0	0	0	1
7	Bilophila (AC)	10	12	10	12	12	11	0	0	0	0	0	0	2	0	0	0	0	1
8	Blautia (AC)	10	12	10	12	12	11	0	0	0	0	0	0	2	0	0	0	0	1
9	Butyricicoccus (AC)	10	12	10	12	12	11	0	0	0	0	0	0	2	0	0	0	0	1
10	Butyricimonas (AC)	10	12	10	12	12	11	0	0	0	0	0	0	2	0	0	0	0	1
11	Campylobacter (AC)	10	12	10	12	12	11	0	0	0	0	0	0	2	0	0	0	0	1
12	Catenibacterium (AC)	10	12	10	12	12	11	0	0	0	0	0	0	2	0	0	0	0	1
13	Clostridium (AC)	10	12	10	12	12	11	0	0	0	0	0	0	2	0	0	0	0	1
14	Collinsella (AC)	10	12	10	12	12	11	0	0	0	0	0	0	2	0	0	0	0	1
15	Coprococcus (AC)	10	12	10	12	12	11	0	0	0	0	0	0	2	0	0	0	0	1
16	Desulfovibrio (AC)	10	12	10	12	12	11	0	0	0	0	0	0	2	0	0	0	0	1
17	Dialister (AC)	10	12	10	12	12	11	0	0	0	0	0	0	2	0	0	0	0	1
18	Dorea (AC)	10	12	10	12	12	11	0	0	0	0	0	0	2	0	0	0	0	1
19	Escherichia (AC)	10	12	10	12	12	11	0	0	0	0	0	0	2	0	0	0	0	1
20	Eubacterium (AC)	10	12	10	12	12	11	0	0	0	0	0	0	2	0	0	0	0	1
21	Faecalibacterium (AC)	10	12	10	12	12	11	0	0	0	0	0	0	2	0	0	0	0	1
22	Flexispira (AC)	10	12	10	12	12	11	0	0	0	0	0	0	2	0	0	0	0	1
23	Lactobacillus (AC)	10	12	10	12	12	11	0	0	0	0	0	0	2	0	0	0	0	1
24	Lactococcus (AC)	10	12	10	12	12	11	0	0	0	0	0	0	2	0	0	0	0	1
25	Leuconostoc (AC)	10	12	10	12	12	11	0	0	0	0	0	0	2	0	0	0	0	1
26	Megasphaera (AC)	10	12	10	12	12	11	0	0	0	0	0	0	2	0	0	0	0	1
27	Mitsuokella (AC)	10	12	10	12	12	11	0	0	0	0	0	0	2	0	0	0	0	1
28	Mogibacterium (AC)	10	12	10	12	12	11	0	0	0	0	0	0	2	0	0	0	0	1
29	Mucispirillum (AC)	10	12	10	12	12	11	0	0	0	0	0	0	2	0	0	0	0	1
30	Oscillospira (AC)	10	12	10	12	12	11	0	0	0	0	0	0	2	0	0	0	0	1
31	Parabacteroides (AC)	10	12	10	12	12	11	0	0	0	0	0	0	2	0	0	0	0	1
32	Pasteurella (AC)	10	12	10	12	12	11	0	0	0	0	0	0	2	0	0	0	0	1
33	Peptococcus (AC)	10	12	10	12	12	11	0	0	0	0	0	0	2	0	0	0	0	1
34	Phascolarctobacterium (AC)	10	12	10	12	12	11	0	0	0	0	0	0	2	0	0	0	0	1
35	Prevotella (AC)	10	12	10	12	12	11	0	0	0	0	0	0	2	0	0	0	0	1
36	Prevotella (AC)	10	12	10	12	12	11	0	0	0	0	0	0	2	0	0	0	0	1
37	Pyramidobacter (AC)	10	12	10	12	12	11	0	0	0	0	0	0	2	0	0	0	0	1

Table S2. Sample size and missingness.

No	Variable	Sample Size[1]						Not-quantified observations (NQ)						Missing Observations[2]					
		CON	BMOS	BMOS + HMO	HMO	OF	OF + 2'FL	CON	BMOS	BMOS + HMO	HMO	OF	OF + 2'FL	CON	BMOS	BMOS + HMO	HMO	OF	OF + 2'FL
38	RFN20 (AC)	10	12	10	12	12	11	0	0	0	0	0	0	2	0	0	0	0	1
39	Ruminococcus (AC)	10	12	10	12	12	11	0	0	0	0	0	0	2	0	0	0	0	1
40	Ruminococcus (AC)	10	12	10	12	12	11	0	0	0	0	0	0	2	0	0	0	0	1
41	Sharpea (AC)	10	12	10	12	12	11	0	0	0	0	0	0	2	0	0	0	0	1
42	Streptococcus (AC)	10	12	10	12	12	11	0	0	0	0	0	0	2	0	0	0	0	1
43	Sutterella (AC)	10	12	10	12	12	11	0	0	0	0	0	0	2	0	0	0	0	1
44	Synergistes (AC)	10	12	10	12	12	11	0	0	0	0	0	0	2	0	0	0	0	1
45	Unclassified (AC)	10	12	10	12	12	11	0	0	0	0	0	0	2	0	0	0	0	1
46	Turicibacter (AC)	10	12	10	12	12	11	0	0	0	0	0	0	2	0	0	0	0	1
47	Uncl. Coriobacteriaceae (AC)	10	12	10	12	12	11	0	0	0	0	0	0	2	0	0	0	0	1
48	Uncl. Desulfovibrionaceae (AC)	10	12	10	12	12	11	0	0	0	0	0	0	2	0	0	0	0	1
49	Uncl. Elusimicrobiaceae (AC)	10	12	10	12	12	11	0	0	0	0	0	0	2	0	0	0	0	1
50	Uncl. Enterobacteriaceae (AC)	10	12	10	12	12	11	0	0	0	0	0	0	2	0	0	0	0	1
51	Uncl. Enterococcaceae (AC)	10	12	10	12	12	11	0	0	0	0	0	0	2	0	0	0	0	1
52	Uncl. Erysipelotrichaceae (AC)	10	12	10	12	12	11	0	0	0	0	0	0	2	0	0	0	0	1
53	Uncl. Fusobacteriaceae (AC)	10	12	10	12	12	11	0	0	0	0	0	0	2	0	0	0	0	1
54	Uncl. Lachnospiraceae (AC)	10	12	10	12	12	11	0	0	0	0	0	0	2	0	0	0	0	1
55	Uncl. Lactobacillaceae (AC)	10	12	10	12	12	11	0	0	0	0	0	0	2	0	0	0	0	1
56	Uncl. Leuconostocaceae (AC)	10	12	10	12	12	11	0	0	0	0	0	0	2	0	0	0	0	1
57	Uncl. Mogibacteriaceae (AC)	10	12	10	12	12	11	0	0	0	0	0	0	2	0	0	0	0	1
58	Uncl. Paraprevotellaceae (AC)	10	12	10	12	12	11	0	0	0	0	0	0	2	0	0	0	0	1
59	Uncl. Prevotellaceae (AC)	10	12	10	12	12	11	0	0	0	0	0	0	2	0	0	0	0	1
60	Uncl. Rikenellaceae (AC)	10	12	10	12	12	11	0	0	0	0	0	0	2	0	0	0	0	1
61	Uncl. Ruminococcaceae (AC)	10	12	10	12	12	11	0	0	0	0	0	0	2	0	0	0	0	1
62	Uncl. S24-7 (AC)	10	12	10	12	12	11	0	0	0	0	0	0	2	0	0	0	0	1
63	Uncl. Veillonellaceae (AC)	10	12	10	12	12	11	0	0	0	0	0	0	2	0	0	0	0	1
64	Acidaminococcus (F)	10	11	9	11	11	11	0	0	0	0	0	0	2	1	1	1	1	1
65	Akkermansia (F)	10	11	9	11	11	11	0	0	0	0	0	0	2	1	1	1	1	1
66	Alistipes (F)	10	11	9	11	11	11	0	0	0	0	0	0	2	1	1	1	1	1
67	Anaerotruncus (F)	10	11	9	11	11	11	0	0	0	0	0	0	2	1	1	1	1	1
68	Bacteroides (F)	10	11	9	11	11	11	0	0	0	0	0	0	2	1	1	1	1	1
69	Bifidobacterium (F)	10	11	9	11	11	11	0	0	0	0	0	0	2	1	1	1	1	1
70	Bilophila (F)	10	11	9	11	11	11	0	0	0	0	0	0	2	1	1	1	1	1
71	Blautia (F)	10	11	9	11	11	11	0	0	0	0	0	0	2	1	1	1	1	1
72	Butyricicoccus (F)	10	11	9	11	11	11	0	0	0	0	0	0	2	1	1	1	1	1
73	Butyricimonas (F)	10	11	9	11	11	11	0	0	0	0	0	0	2	1	1	1	1	1
74	Campylobacter (F)	10	11	9	11	11	11	0	0	0	0	0	0	2	1	1	1	1	1
75	Catenibacterium (F)	10	11	9	11	11	11	0	0	0	0	0	0	2	1	1	1	1	1
76	Christensenella (F)	10	11	9	11	11	11	0	0	0	0	0	0	2	1	1	1	1	1

Table S2. Sample size and missingness.

No	Variable	Sample Size[1]						Not-quantified observations (NQ)						Missing Observations[2]					
		CON	BMOS	BMOS + HMO	HMO	OF	OF + 2'FL	CON	BMOS	BMOS + HMO	HMO	OF	OF + 2'FL	CON	BMOS	BMOS + HMO	HMO	OF	OF + 2'FL
77	Clostridium (F)	10	11	9	11	11	11	0	0	0	0	0	0	2	1	1	1	1	1
78	Clostridium (F)	10	11	9	11	11	11	0	0	0	0	0	0	2	1	1	1	1	1
79	Collinsella (F)	10	11	9	11	11	11	0	0	0	0	0	0	2	1	1	1	1	1
80	Coprococcus (F)	10	11	9	11	11	11	0	0	0	0	0	0	2	1	1	1	1	1
81	Desulfovibrio (F)	10	11	9	11	11	11	0	0	0	0	0	0	2	1	1	1	1	1
82	Dialister (F)	10	11	9	11	11	11	0	0	0	0	0	0	2	1	1	1	1	1
83	Dorea (F)	10	11	9	11	11	11	0	0	0	0	0	0	2	1	1	1	1	1
84	Escherichia (F)	10	11	9	11	11	11	0	0	0	0	0	0	2	1	1	1	1	1
85	Eubacterium (F)	10	11	9	11	11	11	0	0	0	0	0	0	2	1	1	1	1	1
86	Faecalibacterium (F)	10	11	9	11	11	11	0	0	0	0	0	0	2	1	1	1	1	1
87	Flexispira (F)	10	11	9	11	11	11	0	0	0	0	0	0	2	1	1	1	1	1
88	Lactobacillus (F)	10	11	9	11	11	11	0	0	0	0	0	0	2	1	1	1	1	1
89	Lactococcus (F)	10	11	9	11	11	11	0	0	0	0	0	0	2	1	1	1	1	1
90	Leuconostoc (F)	10	11	9	11	11	11	0	0	0	0	0	0	2	1	1	1	1	1
91	Megasphaera (F)	10	11	9	11	11	11	0	0	0	0	0	0	2	1	1	1	1	1
92	Mitsuokella (F)	10	11	9	11	11	11	0	0	0	0	0	0	2	1	1	1	1	1
93	Oscillospira (F)	10	11	9	11	11	11	0	0	0	0	0	0	2	1	1	1	1	1
94	p-75-a5 (F)	10	11	9	11	11	11	0	0	0	0	0	0	2	1	1	1	1	1
95	Parabacteroides (F)	10	11	9	11	11	11	0	0	0	0	0	0	2	1	1	1	1	1
96	Phascolarctobacterium (F)	10	11	9	11	11	11	0	0	0	0	0	0	2	1	1	1	1	1
97	Prevotella (F)	10	11	9	11	11	11	0	0	0	0	0	0	2	1	1	1	1	1
98	Prevotella (F)	10	11	9	11	11	11	0	0	0	0	0	0	2	1	1	1	1	1
99	RFN20 (F)	10	11	9	11	11	11	0	0	0	0	0	0	2	1	1	1	1	1
100	Ruminococcus (F)	10	11	9	11	11	11	0	0	0	0	0	0	2	1	1	1	1	1
101	Ruminococcus (F)	10	11	9	11	11	11	0	0	0	0	0	0	2	1	1	1	1	1
102	Sharpea (F)	10	11	9	11	11	11	0	0	0	0	0	0	2	1	1	1	1	1
103	Succiniclasticum (F)	10	11	9	11	11	11	0	0	0	0	0	0	2	1	1	1	1	1
104	Sutterella (F)	10	11	9	11	11	11	0	0	0	0	0	0	2	1	1	1	1	1
105	Synergistes (F)	10	11	9	11	11	11	0	0	0	0	0	0	2	1	1	1	1	1
106	Turicibacter (F)	10	11	9	11	11	11	0	0	0	0	0	0	2	1	1	1	1	1
107	Uncl. Barnesiellaceae (F)	10	11	9	11	11	11	0	0	0	0	0	0	2	1	1	1	1	1
108	Uncl. Christensenellaceae (F)	10	11	9	11	11	11	0	0	0	0	0	0	2	1	1	1	1	1
109	Uncl. Coriobacteriaceae (F)	10	11	9	11	11	11	0	0	0	0	0	0	2	1	1	1	1	1
110	Uncl. Desulfovibrionaceae (F)	10	11	9	11	11	11	0	0	0	0	0	0	2	1	1	1	1	1
111	Uncl. Elusimicrobiaceae (F)	10	11	9	11	11	11	0	0	0	0	0	0	2	1	1	1	1	1
112	Uncl. Enterobacteriaceae (F)	10	11	9	11	11	11	0	0	0	0	0	0	2	1	1	1	1	1
113	Uncl. Erysipelotrichaceae (F)	10	11	9	11	11	11	0	0	0	0	0	0	2	1	1	1	1	1
114	Uncl. Fusobacteriaceae (F)	10	11	9	11	11	11	0	0	0	0	0	0	2	1	1	1	1	1
115	Uncl. Lachnospiraceae (F)	10	11	9	11	11	11	0	0	0	0	0	0	2	1	1	1	1	1

Table S2. Sample size and missingness.

No	Variable	Sample Size[1]						Not-quantified observations (NQ)						Missing Observations[2]					
		CON	BMOS	BMOS + HMO	HMO	OF	OF + 2'FL	CON	BMOS	BMOS + HMO	HMO	OF	OF + 2'FL	CON	BMOS	BMOS + HMO	HMO	OF	OF + 2'FL
116	Uncl. Lactobacillaceae (F)	10	11	9	11	11	11	0	0	0	0	0	0	2	1	1	1	1	1
117	Uncl. Mogibacteriaceae (F)	10	11	9	11	11	11	0	0	0	0	0	0	2	1	1	1	1	1
118	Uncl. Paraprevotellaceae (F)	10	11	9	11	11	11	0	0	0	0	0	0	2	1	1	1	1	1
119	Uncl. Peptostreptococcaceae (F)	10	11	9	11	11	11	0	0	0	0	0	0	2	1	1	1	1	1
120	Uncl. Prevotellaceae (F)	10	11	9	11	11	11	0	0	0	0	0	0	2	1	1	1	1	1
121	Uncl. Rikenellaceae (F)	10	11	9	11	11	11	0	0	0	0	0	0	2	1	1	1	1	1
122	Uncl. Ruminococcaceae (F)	10	11	9	11	11	11	0	0	0	0	0	0	2	1	1	1	1	1
123	Uncl. S24-7 (F)	10	11	9	11	11	11	0	0	0	0	0	0	2	1	1	1	1	1
124	Uncl. Synergistaceae (F)	10	11	9	11	11	11	0	0	0	0	0	0	2	1	1	1	1	1
125	Uncl. Veillonellaceae (F)	10	11	9	11	11	11	0	0	0	0	0	0	2	1	1	1	1	1
126	Uncl. Victivallaceae (F)	10	11	9	11	11	11	0	0	0	0	0	0	2	1	1	1	1	1
127	Latency to first visit (1h)	11	11	10	12	12	12	0	1	0	0	0	0	1	0	0	0	0	0
128	Latency to first visit (48h)	11	11	10	12	12	11	0	1	0	0	0	1	1	0	0	0	0	0
129	Mean visit time (1h)	11	11	10	12	12	12	0	1	0	0	0	0	1	0	0	0	0	0
130	Mean visit time (48h)	11	11	10	12	12	11	0	1	0	0	0	1	1	0	0	0	0	0
131	Nov ave visit time (1h)	10	11	10	11	12	11	1	1	0	1	0	1	1	0	0	0	0	0
132	Nov ave visit time (48h)	10	11	10	11	12	11	1	1	0	1	0	1	1	0	0	0	0	0
133	Nov latency to first visit (1h)	10	11	10	11	12	11	1	1	0	1	0	1	1	0	0	0	0	0
134	Nov latency to first visit (48h)	10	11	10	11	12	11	1	1	0	1	0	1	1	0	0	0	0	0
135	Nov no. of visits (1h)	11	12	10	12	12	12	0	0	0	0	0	0	1	0	0	0	0	0
136	Nov no. of visits (48h)	11	12	10	12	12	12	0	0	0	0	0	0	1	0	0	0	0	0
137	Nov visit time (1h)	11	12	10	12	12	12	0	0	0	0	0	0	1	0	0	0	0	0
138	Nov visit time (48h)	11	12	10	12	12	12	0	0	0	0	0	0	1	0	0	0	0	0
139	Perimeter.p (1h)	11	12	10	12	12	12	0	0	0	0	0	0	1	0	0	0	0	0
140	Perimeter.p (48h)	11	12	10	12	12	12	0	0	0	0	0	0	1	0	0	0	0	0
141	RI (1h)	11	11	10	12	12	12	0	1	0	0	0	0	1	0	0	0	0	0
142	RI (48h)	11	11	10	12	12	11	0	1	0	0	0	1	1	0	0	0	0	0
143	Sam ave visit time (1h)	10	10	10	12	12	11	1	2	0	0	0	1	1	0	0	0	0	0
144	Sam ave visit time (48h)	11	11	10	12	12	11	0	1	0	0	0	1	1	0	0	0	0	0
145	Sam latency to first visit (1h)	10	10	10	12	12	11	1	2	0	0	0	1	1	0	0	0	0	0
146	Sam latency to first visit (48h)	11	11	10	12	12	11	0	1	0	0	0	1	1	0	0	0	0	0
147	Sam no. of visits (1h)	11	12	10	12	12	12	0	0	0	0	0	0	1	0	0	0	0	0
148	Sam no. of visits (48h)	11	12	10	12	12	11	0	0	0	0	0	1	1	0	0	0	0	0
149	Sam visit time (1h)	11	12	10	12	12	12	0	0	0	0	0	0	1	0	0	0	0	0
150	Sam visit time (48h)	11	12	10	12	12	12	0	0	0	0	0	0	1	0	0	0	0	0
151	Total dis. moved (1h)	11	12	10	12	12	12	0	0	0	0	0	0	1	0	0	0	0	0
152	Total dis. moved (48h)	11	12	10	12	12	12	0	0	0	0	0	0	1	0	0	0	0	0
153	Total no. of visits (1h)	11	12	10	12	12	12	0	0	0	0	0	0	1	0	0	0	0	0
154	Total no. of visits (48h)	11	12	10	12	12	12	0	0	0	0	0	0	1	0	0	0	0	0

Table S2. Sample size and missingness.

		Sample Size[1]						Not-quantified observations (NQ)						Missing Observations[2]					
No	Variable	CON	BMOS	BMOS + HMO	HMO	OF	OF + 2'FL	CON	BMOS	BMOS + HMO	HMO	OF	OF + 2'FL	CON	BMOS	BMOS + HMO	HMO	OF	OF + 2'FL
155	Total visit time (1h)	11	12	10	12	12	12	0	0	0	0	0	0	1	0	0	0	0	0
156	Total visit time (48h)	11	12	10	12	12	12	0	0	0	0	0	0	1	0	0	0	0	0
157	*BDNF*	12	12	10	12	12	12	0	0	0	0	0	0	0	0	0	0	0	0
158	*C.FOS*	12	12	10	12	12	12	0	0	0	0	0	0	0	0	0	0	0	0
159	*CHRM1*	12	12	10	12	12	12	0	0	0	0	0	0	0	0	0	0	0	0
160	*CHRM2*	12	12	10	12	12	12	0	0	0	0	0	0	0	0	0	0	0	0
161	*CHRM3*	12	12	10	12	12	12	0	0	0	0	0	0	0	0	0	0	0	0
162	*CHRM5*	12	12	10	12	12	12	0	0	0	0	0	0	0	0	0	0	0	0
163	*CHRNA2*	12	12	10	12	12	12	0	0	0	0	0	0	0	0	0	0	0	0
164	*CHRNA7*	12	12	10	12	12	12	0	0	0	0	0	0	0	0	0	0	0	0
165	*CHRNB4*	12	12	10	12	12	12	0	0	0	0	0	0	0	0	0	0	0	0
166	*CREB*	12	12	10	12	12	12	0	0	0	0	0	0	0	0	0	0	0	0
167	*CREBBP*	12	12	10	12	12	12	0	0	0	0	0	0	0	0	0	0	0	0
168	*DLG4*	12	12	10	12	12	12	0	0	0	0	0	0	0	0	0	0	0	0
169	*EGR1*	12	12	10	12	12	12	0	0	0	0	0	0	0	0	0	0	0	0
170	*GABBR1*	12	12	10	12	12	12	0	0	0	0	0	0	0	0	0	0	0	0
171	*GABRA1*	12	12	10	12	12	12	0	0	0	0	0	0	0	0	0	0	0	0
172	*GABRA2*	12	12	10	12	12	12	0	0	0	0	0	0	0	0	0	0	0	0
173	*GABRA5*	12	12	10	12	12	12	0	0	0	0	0	0	0	0	0	0	0	0
174	*GABRB2*	12	12	10	12	12	12	0	0	0	0	0	0	0	0	0	0	0	0
175	*GABRD*	12	12	10	12	12	12	0	0	0	0	0	0	0	0	0	0	0	0
176	*GABRG2*	12	12	10	12	12	12	0	0	0	0	0	0	0	0	0	0	0	0
177	*GABRR1*	12	12	10	12	12	12	0	0	0	0	0	0	0	0	0	0	0	0
178	*GAD*	12	12	10	12	12	12	0	0	0	0	0	0	0	0	0	0	0	0
179	*GLRA1*	12	12	10	12	12	12	0	0	0	0	0	0	0	0	0	0	0	0
180	*GLRA2*	12	12	10	12	12	12	0	0	0	0	0	0	0	0	0	0	0	0
181	*GLRA3*	12	12	10	12	12	12	0	0	0	0	0	0	0	0	0	0	0	0
182	*GRIA1*	12	12	10	12	12	12	0	0	0	0	0	0	0	0	0	0	0	0
183	*GRIA2*	12	12	10	12	12	12	0	0	0	0	0	0	0	0	0	0	0	0
184	*GRIA3*	12	12	10	12	12	12	0	0	0	0	0	0	0	0	0	0	0	0
185	*GRIA4*	12	12	10	12	12	12	0	0	0	0	0	0	0	0	0	0	0	0
186	*GRIN1*	12	12	10	12	12	12	0	0	0	0	0	0	0	0	0	0	0	0
187	*GRIN2A*	12	12	10	12	12	12	0	0	0	0	0	0	0	0	0	0	0	0
188	*GRIN2B*	12	12	10	12	12	12	0	0	0	0	0	0	0	0	0	0	0	0
189	*GRIN2D*	12	12	10	12	12	12	0	0	0	0	0	0	0	0	0	0	0	0
190	*HDAC1*	12	12	10	12	12	12	0	0	0	0	0	0	0	0	0	0	0	0
191	*HDAC2*	12	12	10	12	12	12	0	0	0	0	0	0	0	0	0	0	0	0
192	*HDAC3*	12	12	10	12	12	12	0	0	0	0	0	0	0	0	0	0	0	0
193	*HDAC4*	12	12	10	12	12	12	0	0	0	0	0	0	0	0	0	0	0	0

Table S2. Sample size and missingness.

		Sample Size[1]						Not-quantified observations (NQ)						Missing Observations[2]					
**No**	**Variable**	**CON**	**BMOS**	**BMOS + HMO**	**HMO**	**OF**	**OF + 2'FL**	**CON**	**BMOS**	**BMOS + HMO**	**HMO**	**OF**	**OF + 2'FL**	**CON**	**BMOS**	**BMOS + HMO**	**HMO**	**OF**	**OF + 2'FL**
194	*HDAC5*	12	12	10	12	12	12	0	0	0	0	0	0	0	0	0	0	0	0
195	*HDAC7*	12	12	10	12	12	12	0	0	0	0	0	0	0	0	0	0	0	0
196	*HDAC8*	12	12	10	12	12	12	0	0	0	0	0	0	0	0	0	0	0	0
197	*HDAC9*	12	12	10	12	12	12	0	0	0	0	0	0	0	0	0	0	0	0
198	*HOMER1*	12	12	10	12	12	12	0	0	0	0	0	0	0	0	0	0	0	0
199	*IGF1*	12	12	10	12	12	12	0	0	0	0	0	0	0	0	0	0	0	0
200	*IGF2*	12	12	10	12	12	12	0	0	0	0	0	0	0	0	0	0	0	0
201	*MAG*	12	12	10	12	12	12	0	0	0	0	0	0	0	0	0	0	0	0
202	*MBP*	12	12	10	12	12	12	0	0	0	0	0	0	0	0	0	0	0	0
203	*NCAM1*	12	12	10	12	12	12	0	0	0	0	0	0	0	0	0	0	0	0
204	*NPY*	12	12	10	12	12	12	0	0	0	0	0	0	0	0	0	0	0	0
205	*NR3C1*	12	12	10	12	12	12	0	0	0	0	0	0	0	0	0	0	0	0
206	*NR3C2*	12	12	10	12	12	12	0	0	0	0	0	0	0	0	0	0	0	0
207	*NR4A1*	12	12	10	12	12	12	0	0	0	0	0	0	0	0	0	0	0	0
208	*NR4A2*	12	12	10	12	12	12	0	0	0	0	0	0	0	0	0	0	0	0
209	*PLP*	12	12	10	12	12	12	0	0	0	0	0	0	0	0	0	0	0	0
210	*PP1AC*	12	12	10	12	12	12	0	0	0	0	0	0	0	0	0	0	0	0
211	*SIRT1*	12	12	10	12	12	12	0	0	0	0	0	0	0	0	0	0	0	0
212	*SLC17A6*	12	12	10	12	12	12	0	0	0	0	0	0	0	0	0	0	0	0
213	*SLC17A7*	12	12	10	12	12	12	0	0	0	0	0	0	0	0	0	0	0	0
214	*SLC17A8*	12	12	10	12	12	12	0	0	0	0	0	0	0	0	0	0	0	0
215	*SLC1A1*	12	12	10	12	12	12	0	0	0	0	0	0	0	0	0	0	0	0
216	*SLC1A2*	12	12	10	12	12	12	0	0	0	0	0	0	0	0	0	0	0	0
217	*SLC1A3*	12	12	10	12	12	12	0	0	0	0	0	0	0	0	0	0	0	0
218	*SLC1A6*	12	12	10	12	12	12	0	0	0	0	0	0	0	0	0	0	0	0
219	*SLC32A1*	12	12	10	12	12	12	0	0	0	0	0	0	0	0	0	0	0	0
220	*SLC6A1*	12	12	10	12	12	12	0	0	0	0	0	0	0	0	0	0	0	0
221	*SLC6A11*	12	12	10	12	12	12	0	0	0	0	0	0	0	0	0	0	0	0
222	*SLC6A13*	12	12	10	12	12	12	0	0	0	0	0	0	0	0	0	0	0	0
223	*SNAP25*	12	12	10	12	12	12	0	0	0	0	0	0	0	0	0	0	0	0
224	*SYP*	12	12	10	12	12	12	0	0	0	0	0	0	0	0	0	0	0	0
225	*UBE3A*	12	12	10	12	12	12	0	0	0	0	0	0	0	0	0	0	0	0
226	*5-HTR1*	12	12	10	12	12	12	0	0	0	0	0	0	0	0	0	0	0	0
227	*5-HTR2*	12	12	10	12	12	12	0	0	0	0	0	0	0	0	0	0	0	0
228	*5-HTR4*	12	12	10	12	12	12	0	0	0	0	0	0	0	0	0	0	0	0
229	*5-HTR7*	12	12	10	12	12	12	0	0	0	0	0	0	0	0	0	0	0	0
230	GABA	11	8	9	11	10	12	0	0	0	0	0	0	1	4	1	1	2	0
231	Glutathione	9	8	9	10	10	12	2	0	0	1	0	0	1	4	1	1	2	0
232	Inositol	10	8	9	10	10	12	1	0	0	1	0	0	1	4	1	1	2	0

Table S2. Sample size and missingness.

No	Variable	Sample Size[1]						Not-quantified observations (NQ)						Missing Observations[2]					
		CON	BMOS	BMOS + HMO	HMO	OF	OF + 2'FL	CON	BMOS	BMOS + HMO	HMO	OF	OF + 2'FL	CON	BMOS	BMOS + HMO	HMO	OF	OF + 2'FL
233	N-acetylaspartate	10	8	9	10	10	12	1	0	0	1	0	0	1	4	1	1	2	0
234	faCaudate	11	12	9	10	11	10	0	0	0	0	0	0	1	0	1	2	1	2
235	faCorpus C.	11	12	9	10	11	10	0	0	0	0	0	0	1	0	1	2	1	2
236	faCerebellum	11	12	9	10	11	10	0	0	0	0	0	0	1	0	1	2	1	2
237	faInternal C.	11	12	9	10	11	10	0	0	0	0	0	0	1	0	1	2	1	2
238	faLeft Crtx	11	12	9	10	11	10	0	0	0	0	0	0	1	0	1	2	1	2
239	faLeft Hip.	10	12	9	10	11	10	1	0	0	0	0	0	1	0	1	2	1	2
240	faRight Crtx	11	12	9	10	11	10	0	0	0	0	0	0	1	0	1	2	1	2
241	faRight Hip.	11	12	9	10	11	10	0	0	0	0	0	0	1	0	1	2	1	2
242	faThalamus	11	12	9	10	11	10	0	0	0	0	0	0	1	0	1	2	1	2
243	faWhole B.	11	12	9	10	11	10	0	0	0	0	0	0	1	0	1	2	1	2
244	faWhite	11	12	9	10	11	10	0	0	0	0	0	0	1	0	1	2	1	2
245	Cerebral Aqueduct	11	12	9	10	12	11	0	0	0	0	0	0	1	0	1	2	0	1
246	Caudate	11	12	9	10	12	11	0	0	0	0	0	0	1	0	1	2	0	1
247	Corpus C.	11	12	9	10	12	11	0	0	0	0	0	0	1	0	1	2	0	1
248	Cerebellum	11	12	9	10	12	11	0	0	0	0	0	0	1	0	1	2	0	1
249	CSF	11	12	9	10	12	11	0	0	0	0	0	0	1	0	1	2	0	1
250	Fourth Vent.	11	12	9	10	12	11	0	0	0	0	0	0	1	0	1	2	0	1
251	Grey M.	11	12	9	10	12	11	0	0	0	0	0	0	1	0	1	2	0	1
252	Hypothalamus	11	12	9	10	12	11	0	0	0	0	0	0	1	0	1	2	0	1
253	Internal C.	11	12	9	10	12	11	0	0	0	0	0	0	1	0	1	2	0	1
254	Left Crtx	11	12	9	10	12	11	0	0	0	0	0	0	1	0	1	2	0	1
255	Left Hip.	11	12	9	10	12	11	0	0	0	0	0	0	1	0	1	2	0	1
256	Lateral Vent.	11	12	9	10	12	11	0	0	0	0	0	0	1	0	1	2	0	1
257	Medul	11	12	9	10	12	11	0	0	0	0	0	0	1	0	1	2	0	1
258	Midbr	11	12	9	10	12	11	0	0	0	0	0	0	1	0	1	2	0	1
259	OlfBul	11	12	9	10	12	11	0	0	0	0	0	0	1	0	1	2	0	1
260	Pons	11	12	9	10	12	11	0	0	0	0	0	0	1	0	1	2	0	1
261	PutGP	11	12	9	10	12	11	0	0	0	0	0	0	1	0	1	2	0	1
262	Right Crtx	11	12	9	10	12	11	0	0	0	0	0	0	1	0	1	2	0	1
263	Right Hip.	11	12	9	10	12	11	0	0	0	0	0	0	1	0	1	2	0	1
264	Thalamus	11	12	9	10	12	11	0	0	0	0	0	0	1	0	1	2	0	1
265	Third Vent.	11	12	9	10	12	11	0	0	0	0	0	0	1	0	1	2	0	1
266	Whole B.	11	12	9	10	12	11	0	0	0	0	0	0	1	0	1	2	0	1
267	White M.	11	12	9	10	12	11	0	0	0	0	0	0	1	0	1	2	0	1
268	Rel. Cer. Aq.	11	12	9	10	12	11	0	0	0	0	0	0	1	0	1	2	0	1
269	Rel. Caudate	11	12	9	10	12	11	0	0	0	0	0	0	1	0	1	2	0	1
270	Rel. Corpus C.	11	12	9	10	12	11	0	0	0	0	0	0	1	0	1	2	0	1
271	Rel. Cerebellum	11	12	9	10	12	11	0	0	0	0	0	0	1	0	1	2	0	1

Table S2. Sample size and missingness.

No	Variable	Sample Size[1]						Not-quantified observations (NQ)						Missing Observations[2]					
		CON	BMOS	BMOS + HMO	HMO	OF	OF + 2'FL	CON	BMOS	BMOS + HMO	HMO	OF	OF + 2'FL	CON	BMOS	BMOS + HMO	HMO	OF	OF + 2'FL
272	Rel. CSF	11	12	9	10	12	11	0	0	0	0	0	0	1	0	1	2	0	1
273	Rel. FV	11	12	9	10	12	11	0	0	0	0	0	0	1	0	1	2	0	1
274	Rel. Grey M.	11	12	9	10	12	11	0	0	0	0	0	0	1	0	1	2	0	1
275	Rel. Hypothalamus	11	12	9	10	12	11	0	0	0	0	0	0	1	0	1	2	0	1
276	Rel. Internal C.	11	12	9	10	12	11	0	0	0	0	0	0	1	0	1	2	0	1
277	Rel. Left Crtx	11	12	9	10	12	11	0	0	0	0	0	0	1	0	1	2	0	1
278	Rel. Left Hip.	11	12	9	10	12	11	0	0	0	0	0	0	1	0	1	2	0	1
279	Rel. Lateral Vent.	11	12	9	10	12	11	0	0	0	0	0	0	1	0	1	2	0	1
280	Rel. Medulla	11	12	9	10	12	11	0	0	0	0	0	0	1	0	1	2	0	1
281	Rel. Midbrain	11	12	9	10	12	11	0	0	0	0	0	0	1	0	1	2	0	1
282	Rel. OlfBul	11	12	9	10	12	11	0	0	0	0	0	0	1	0	1	2	0	1
283	Rel. Pons	11	12	9	10	12	11	0	0	0	0	0	0	1	0	1	2	0	1
284	Rel. PutGP	11	12	9	10	12	11	0	0	0	0	0	0	1	0	1	2	0	1
285	Rel. Right Crtx	11	12	9	10	12	11	0	0	0	0	0	0	1	0	1	2	0	1
286	Rel. Right Hip.	11	12	9	10	12	11	0	0	0	0	0	0	1	0	1	2	0	1
287	Rel. Thalamus	11	12	9	10	12	11	0	0	0	0	0	0	1	0	1	2	0	1
288	Rel. Third Vent.	11	12	9	10	12	11	0	0	0	0	0	0	1	0	1	2	0	1
289	Rel. White M.	11	12	9	10	12	11	0	0	0	0	0	0	1	0	1	2	0	1

[1]Represents the effective sample size used for analysis after cleaning and imputing procedures. [2]Missing observations were imputed with the median value of that group for each respective variable. Abbreviations: CON, control group; HMO, pigs fed human milk oligosaccharides; BMOS, pigs fed bovine milk oligosaccharides; BMOS + HMO, pigs fed both human and bovine milk oligosaccharides; OF, pigs feds oligofructose; OF + 2′-FL, pigs fed oligofructose and 2′-fucosyllactose.

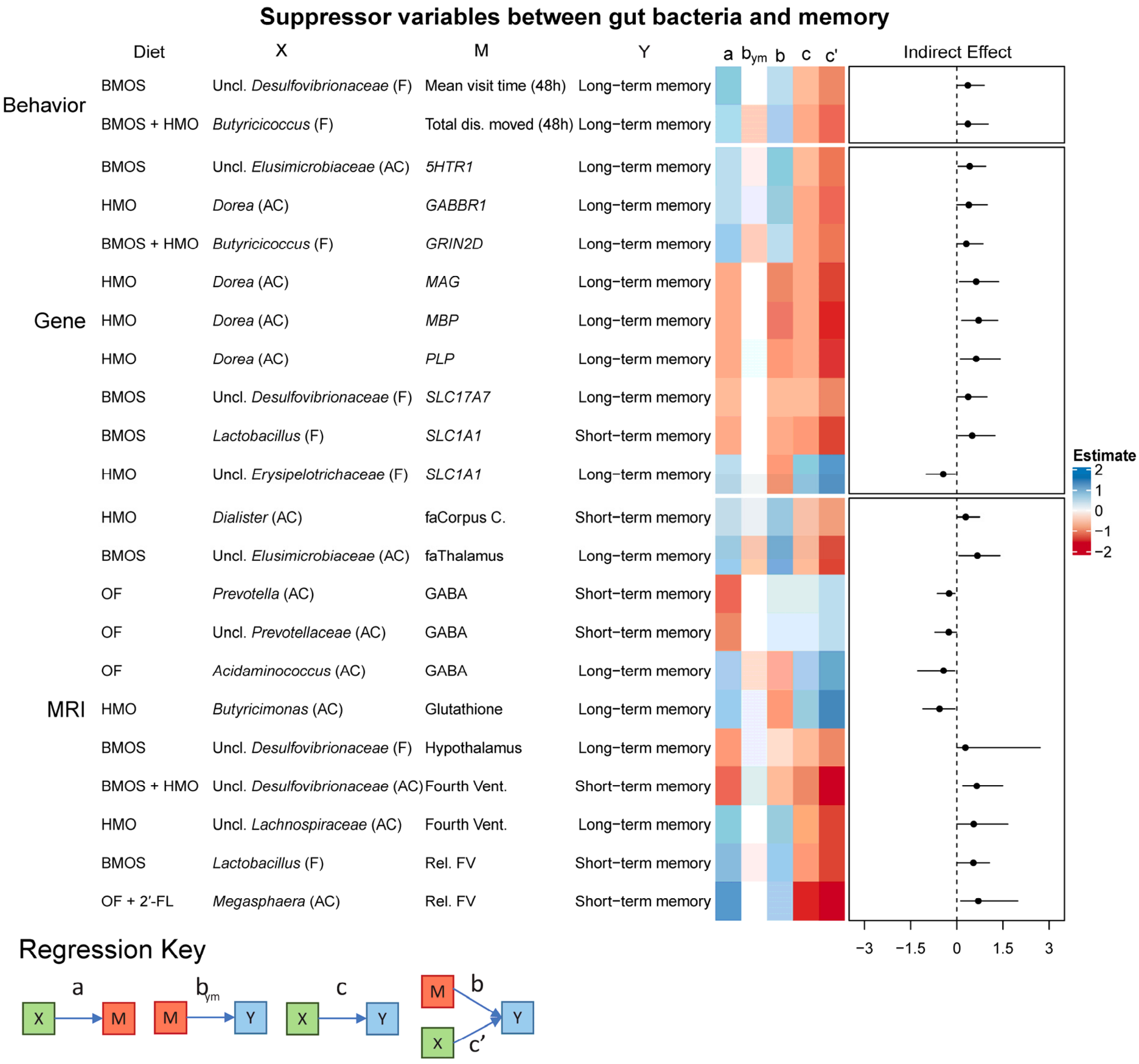

Figure S1. Heatmaps demonstrating suppressor variables organized by mediator type. All mediating variables shown have little-to-no direct relationship to the predicted variable (b_{ym}) but increase the relationship between bacterial genera and memory when included in the model. Only mediations whose confidence intervals of the indirect effect do not include zero are shown. The right-most box represents the estimates of the indirect effect (c-c′) bounded by its lower and upper 95% confidence intervals. Paths a, b_{ym}, b, c, and c′ are the regression coefficients for X → M, M →Y, X → Y, and X + M → Y, respectively. Abbreviations: Uncl., unclassified; CON, control group; HMO, pigs fed human milk oligosaccharides; BMOS, pigs fed bovine milk oligosaccharides; BMOS + HMO, pigs fed both human and bovine milk oligosaccharides; OF, pigs feds oligofructose; OF + 2′-FL, pigs fed oligofructose and 2′-fucosyllactose.
